# OP30 Integrating Real-World Evidence Into Health Technology Assessment In The Americas Region: A Collaborative Initiative By RedETSA

**DOI:** 10.1017/S0266462325100883

**Published:** 2025-12-29

**Authors:** Sebastian Garcia Marti, Guadalupe Montero, Graciela Fernandez, Carlos Rezende, Maricela Curisinche, Mariana Beatriz Pineda-López

## Abstract

**Introduction:**

The Health Technology Assessment Network of the Americas (RedETSA) working groups gather members aiming to develop projects and research jointly. The Real-World Evidence (RWE) Working Group seeks to identify methodologies and approaches allowing RWE integration into health technology assessment (HTA) in the region. This collaborative initiative is crucial to enhancing evidence-based decision-making processes in the Americas, shaping regional health policies.

**Methods:**

A survey was conducted among RedETSA members to identify RWE use in the context of HTA within the network. The survey included responses from 22 members, reflecting diverse perspectives from across the Americas. A search of official documents issued by various regulatory and HTA agencies was carried out to perform a detailed analysis of the RWE frameworks accessible online. Relevant documents published between 2013 and 2024 were selected, reviewing both the official portals of each agency and publicly accessible academic databases. Documents in English, Spanish, Portuguese, and French were searched.

**Results:**

RWE documents from various HTA or regulatory agencies from different countries or regions were identified. The following domains were analyzed from each document: institution type, RWE definition, objective or purpose of RWE use, data quality and tools to assess RWE, data sources used, proposals for analytical methodologies, approved regulatory uses, and specific initiatives or tools. The RWE Working Group document was developed in Spanish. The document provides practical guidelines and checklists for implementing RWE, making it accessible for both specialists and general stakeholders within RedETSA.

**Conclusions:**

The document was a helpful tool aimed at RedETSA members integrating RWE into HTA. Additional tools will include a procedure to meet regulatory and HTA expectations; and a decision tool to support a particular study design adoption. By leveraging these tools, decision-makers in the region can justify their choice of study design and align with regulatory requirements more effectively.

